# Complete genome sequence of *Burkholderia cenocepacia* bacteriophage Karil-mokiny-1

**DOI:** 10.1128/mra.00306-25

**Published:** 2025-07-22

**Authors:** Jack S. Canning, Kak-Ming Ling, Daniel R. Laucirica, Joshua J. Iszatt, Stephen M. Stick, Anthony Kicic, on behalf of Phage WA

**Affiliations:** 1 Wal-Yan Respiratory Research Centre, Kids Research Institute Australia, The University of Western Australia 2720, Perth, Western Australia, Australia; 2 School of Biomedical Sciences, University of Western Australia 2720, Perth, Western Australia, Australia; 3 Occupation, Environment and Safety, School of Population Health, Curtin University 1649, Perth, Western Australia, Australia; 4 Department of Respiratory and Sleep Medicine, Perth Children's Hospital 60081, Nedlands, Western Australia, Australia; 5 Centre for Cell Therapy and Regenerative Medicine, School of Medicine and Pharmacology, The University of Western Australia and Harry Perkins Institute of Medical Research 102804, Perth, Western Australia, Australia; Loyola University Chicago, Chicago, Illinois, USA

**Keywords:** *Burkholderia cepacia *complex, bacteriophage, antimicrobial resistance, genome, cystic fibrosis

## Abstract

*Burkholderia cepacia* complex causes life-threatening respiratory infections. Here, a bacteriophage with activity against *B. cenocepacia* was isolated from wastewater. It has a genome size of 70,144 bp and has the taxonomic classification *Irusalimvirus*. It has no genes associated with lysogeny, bacterial resistance, or virulence.

## ANNOUNCEMENT

*Burkholderia cepacia* complex (BCC) is a grouping of 24 different species from the *Burkholderia* genus (1). BCC is an opportunistic human pathogen known for causing a wide array of infections in immunocompromised individuals (2), most notably respiratory infections in people with cystic fibrosis (CF) (3). This burden is exacerbated by BCC displaying intrinsic antimicrobial resistance (AMR), which limits antibiotic efficacy and complicates treatment (3). In the pursuit of alternative therapies to treat BCC infections, bacteriophages (phages) have seen renewed interest (3). Here, we report the genome sequence of Karil-mokiny-1, a phage isolated using a clinical isolate of *Burkholderia cenocepacia*. Karil-mokiny-kep-djiraly-karakaata-Wadjak (Karil-mokiny-1) was isolated from 5 mL of wastewater collected in Perth, Western Australia (“31.955839 S 115.793828 E”) following a 48-hour enrichment in ½ strength Luria-Bertani (LB) broth at 30°C, and was then triple-plaque purified using the double agar overlay method (4). Karil-mokiny-1 was propagated using a ½ strength Luria-Bertani (LB) overlay at 30°C according to a previously described “scrape” method and stored at 4°C in SM buffer (5). Phage DNA was extracted using the Qiagen (Hilden, Germany) DNeasy blood and tissue kit. High-titer phage lysate was treated with DNase I and RNase A to remove exogenous nucleic acids, and then treated with proteinase K for capsid destruction and release of phage DNA. Phage DNA was then purified in a DNeasy spin column and eluted into nuclease-free water (6). Samples were then sent to the Australian Genome Research Facility (Victoria, Australia), where DNA libraries were created using the NExtera XT kit (Illumina, San Diego, CA, USA), and whole-genome sequencing was performed using the Illumina NovaSeq 6000 (Illumina) platform. Sequencing generated 1,868,306 paired-end reads at 150 bps in length for assembly. Adapters and low-quality reads were removed using BBmap (v39.06) (HEADCROP:10) (7), after which reads were deduplicated, normalized (min = 5 target = 200), and merged. *De novo* assembly was completed using SPAdes (v 3.15.5) (-t 12 m 5 –only-assembler –careful -k 55,77,99,127) (8), and completeness was determined using CheckV (v1.0.3) (9). Read quality control and assembly as described above was completed using Phanta (https://github.com/JoshuaIszatt/phanta, v1.0.3). Annotations were completed using Pharokka (v1.14.6) with the Prokaryotic Virus Remote Homologous Groups (PHROGS) database (10). Antibiotic resistance and bacterial virulence genes were identified from the CARD (11) and VFDB databases, respectively (12), and t-RNAs were determined using ARAGON (11). Taxonomic assignment was completed using VIRIDIC (13), which determined the average nucleotide identity (ANI%) of Karil-mokiny-1 to its closest relatives (https://rhea.icbm.uni-oldenburg.de/viridic/). Default parameters were used for all software unless otherwise specified.

Karil-mokiny-1’s statistics are shown in Table 1. It possesses a coding-complete genome of 70,144 bp in length with a 51.55% GC content. Karil-mokiny-1 shares 70.7%% sequence homology with its closest relative, Burkholderia phage BCSR52, which was classified as a *Bcepfunavirus* (MW460246) at the time of analysis. Due to the low sequence homology between the two phages, we opted to perform ANI% calculations on BCSR52 and found that it shares only 47.9% of its genome with phage BcepF1, the exemplar *Bcepfunavirus* according to the ICTV (NC_009015). Despite being classified as a *Bcepfunavirus*, Karil-mokiny-1 and BCSR52 have significant genomic divergence from other reported phages, which suggests that the two phages likely represent a new viral genus within an unclassified family. Since the acceptance of this publication, BCSR52 has been classified as an *Irusalimvirus,* with which Karil-mokiny-1 now shares a genus.

**TABLE 1:** Genomic characteristics of Karil-mokiny-1

Parameter	Value
Genome size (bp)	70,144
GC content (%)	51.5
Reads in assembly	1,868,306
Depth coverage (normalized reads)	3,476
CheckV quality and completeness score	Complete genome, 99.89%
Coding sequences	122
Hypothetical proteins	88
AMR, virulence, or lysogeny genes	None detected
Closest relative (accession no.)	*Burkholderia* phage BCSR52 (MW460246)
Average nucleotide identity (%)	70.7%

## Data Availability

The complete sequence of Karil-mokiny-1 has been deposited in GenBank under the accession number PV388296 and in the Sequence Read Archive under accession number SRP528998.
